# Bone Evaluation with Micro Finite Element Analysis in Animal Models

**DOI:** 10.3390/tomography11090101

**Published:** 2025-09-01

**Authors:** Behnam Namiranian, Kenichiro Doi, Salem Alenezi, Sameer B. Shah, Saeed Jerban, Eric Y. Chang

**Affiliations:** 1 Department of Radiology, University of California, San Diego, CA 92093, USA; b2namiranian@health.ucsd.edu; 2 Department of Orthopedic Surgery, Faculty of Medicine, Fukuoka University, Fukuoka 810-0180, Japan; m05066kd@gmail.com; 3 Research and Laboratories Sector, Saudi Food and Drug Authority, Riyadh 13513, Saudi Arabia; saenezi@gmail.com; 4 Department of Orthopaedic Surgery, University of California, San Diego, CA 92093, USA; sbshah@health.ucsd.edu; 5 Department of Bioengineering, University of California, San Diego, CA 92093, USA; 6 Research Service, Veterans Affairs San Diego Healthcare System, San Diego, CA 92161, USA; 7 Radiology Service, Veterans Affairs San Diego Healthcare System, San Diego, CA 92161, USA

**Keywords:** micro-computed tomography, micro finite element analysis, bone, sponge bone, mechanical competence

## Abstract

Micro-computed tomography (micro-CT) is a commonly used tool for bone evaluation in animal model research. Micro-scale finite element analysis (µFEA) has been proposed to account for different loading scenarios, detailed three-dimensional (3D) bone structure, material properties, and distribution obtained from micro-CT to estimate bone mechanical properties and to predict its potential fracture. The in vivo application of µFEA has been limited to animal models due to the smaller bore size of micro-CT and the long scan time. This narrative review article describes studies that used micro-CT-based µFEA to predict bone mechanical competence, understand bone fracture and remodeling mechanisms, and to evaluate the impacts of the therapeutics, implants, and surgical interventions. Moreover, the concept, limitations, and future potentials of micro-CT-based FEA are discussed.

## 1. Introduction

There are two main types of bone: cortical (compact or dense) and trabecular (cancellous or spongy). Cortical bone is the dense bone that forms the outer sheath, and it makes up most of the skeleton’s weight [1]. Trabecular bone is engulfed between cortical bone shells to improve energy absorbance capabilities (e.g., in the skull, ribs, and vertebrae) or to distribute loads in the joints at both ends of the long bones (e.g., the femoral head and condyle) [2].

In osteoporosis, a common disease in the elderly, the balance between new bone generation and old bone resorption is impaired, which results in bone mass reduction [3]. Consequently, the skeleton becomes weak, brittle, and susceptible to fracture. Traditionally, a reduction in bone mineral density (BMD) and the deterioration of bone architecture have been considered as the main predictors of fracture risk [4]. The current standard for osteoporosis diagnosis and bone fracture risk assessment is to measure the areal bone mineral density (aBMD, g/cm^2^) by dual-energy X-ray absorptiometry (DXA). However, aBMD is limited in a number of ways, including the lack of consideration of bone geometry and microarchitecture, inability to discern cortical bone from trabecular structure (while both contribute substantially to the bone strength), and the lack of accounting for various loading scenarios and directions [5].

Trabecular bone architecture is traditionally characterized by trabeculae thickness, number, density, connectivity, and intertrabecular space in a two-dimensional (2D) or three-dimensional (3D) fashion, which all play important roles in bone mechanical resilience [6]. The characterization of cortical bone architecture is limited mainly to bone thickness and porosity.

Finite element analysis (FEA) has been proposed to account for different loading scenarios and directions, in addition to the 3D bone structural arrangement, material properties, and distribution, in order to provide a more accurate determination of bone mechanical properties [7]. Finite element (FE) models in the literature can be categorized as, first, homogenized or continuum macro-scale models, in which the bone and marrow structures are merged as a continuous solid volume but with differing material properties, and second, micro-scale models (µFEA) based on high-resolution images, in which the detailed internal bone structure is considered in the model. These two approaches can be complementary depending on the nature of the questions researchers are trying to answer. The macro-scale models are often based on conventional computed tomography (CT) images (200 to 1000 µm voxel sizes), while micro-scale models are often based on high-resolution images from micro-computed tomography (micro-CT) with voxel sizes from ~1 to 100 µm.

Both CT and micro-CT collect 2D X-ray projections of specimens; thereafter, correction and back projection algorithms enable the reconstruction of the 3D volume of the bone specimens [8]. The radiodensity values of the reconstructed voxels can be reported in Hounsfield units (HU) or BMD, which requires calibration and phantom scans [9]. The 3D image resolutions are much higher in micro-CT than in CT due to the finer X-ray detectors and arrays, as well as the much shorter achievable distances between the X-ray detectors and specimens. However, micro-CT bore sizes are much smaller than CT ones (<100 mm versus ~70–90 cm), which limits the in vivo applications to animal models alone due to the smaller sizes and lower concerns about exposure to X-ray radiation. Numerous studies have reported using micro-CT to evaluate bone in animal models by focusing on the trabeculae thickness, number, density, connectivity, and space [10,11], which are limited in predicting bone mechanical properties.

In this review study, we summarize the methods and results of micro-CT-based FEA studies performed on animal models and discuss potential limitations and future directions.

## 2. Materials and Methods

This review was conducted between September 2023 and March 2025 and aimed to describe reported FEA studies performed on animal models based on micro-CT images, in vivo or ex vivo. The literature search was mainly performed in PubMed and Google Scholar databases using the following keywords: “Finite Element,” “Bone,” and “Micro Computed Tomography.” Only studies published after the year 2000 were considered. Titles and abstracts were screened to exclude duplicated records, non-English-language reports, and review articles. Only studies that used animal models were included in this review. This study could not be considered a systematic review, as some of the requirements for the PRISMA guidelines were not fulfilled. For example, searching was limited to only two main databases, some study selections were carried out by only one author, and the numbers of studies in each step of screening were not recorded.

## 3. Mechanical Properties and Concept

The mechanical behavior of a structure is determined by its geometry, material properties, and the loading scenario and direction. A basic understanding of mechanical parameters is required to interpret FEA results, such as stress, strain, elastic modulus, and strength. These basic mechanical parameters are defined and presented schematically in Table 1.

Normal stress (e.g., compressive or tensile stress) is defined as perpendicular to the volume unit surface and equal to the load magnitude per unit area. Shear stress is similar but defined as parallel to the volume unit surface. The international standard unit of stress is the pascal (Pa), which equals 1 newton over 1 square meter (N/m^2^). Strain is defined as the fractional change in the length of a loaded body, which is usually given as a percentage or micro-strain (i.e., 1/1 million). Strain can also be described as normal and shear relative to the spatial orientation of the unit volume.

The elasticity of a material describes its tendency to retain its original size and shape after being subjected to a deforming force or stress. The modulus of elasticity can be quantified as the ratio of stress to strain (units of N/m^2^, or Pa), which is usually in the range of several gigapascals (GPa) in bone. The modulus of elasticity can be quantified depending on the direction of stress and strain. Young’s modulus (i.e., normal modulus), E, quantifies elasticity in the direction normal to the unit volume surface. In contrast, the shear modulus, G, quantifies elasticity in the transverse direction to the unit volume surface [12].

When a sample is compressed, it becomes shorter along the loading direction (negative strain) but broader in the transverse direction (positive strain), and vice versa for extended samples. The negative ratio of the transverse strain component to the longitudinal strain component is called Poisson’s ratio (υ), which equals 0.5 for incompressible fluids but 0.1 to 0.33 for bone [12].

**Table 1:** Definition of the basic mechanical parameters discussed in this study. A portion of this table was published previously by Jerban et al. [13]. Reprinting permission was granted based on Creative Commons use guidelines (https://creativecommons.org/licenses/by/4.0/, (accessed on 1 April 2025)).

Mechanical Parameters	Formula	Definition	Schematics
Normal stress	$\sigma =\frac{F}{A}$	The magnitude of the force applied on the unit area, perpendicular to the force direction	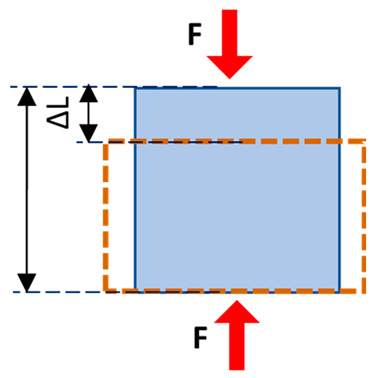
Normal strain	$\varepsilon =\frac{∆L}{L_{0}}$	The change in length of the sample per its original unit length, parallel to the force direction	
Young’s modulus	$E=\frac{\sigma }{\varepsilon }$	Normal stress to normal strain ratio, as the elasticity defined in applied force direction but perpendicular to the unit volume surface	
Shear stress	$\tau =\frac{F}{A}$	The magnitude of the force applied on the unit area, parallel to the force direction	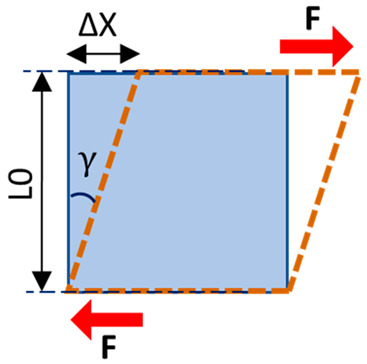
Shear strain	$\gamma =\frac{∆X}{L_{0}}$	The angular change in the original right angles of the unit volume after shear stress application	
Shear modulus	$G=\frac{\tau }{\gamma }$ $G=\frac{E}{2(1+v)}$	Shear stress to shear strain ratio, as the elasticity defined in applied force direction and parallel to the unit volume surface	
Poisson’s ratio	$\upsilon =-\frac{\varepsilon_{\gamma }}{\varepsilon_{x}}$	The negative ratio of the transverse strain component to the longitudinal strain component	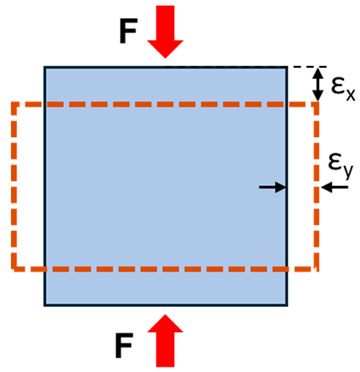

**Figure 1:**
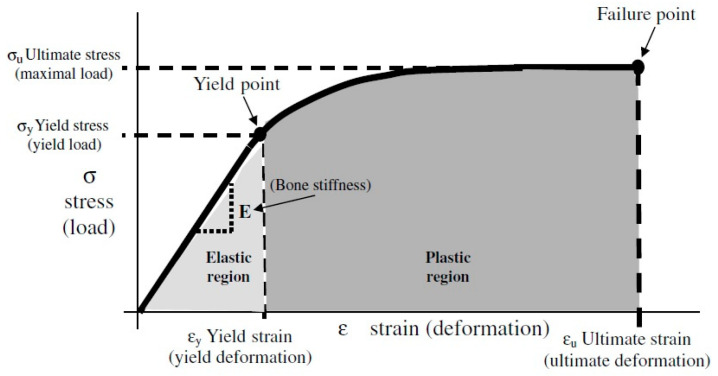
The stress–strain curve obtained by loading a sample of compact bone in tension. This figure was previously presented by Sharir et al. [9]. Reprinting permission was granted through Rightslink system.

The elastic behavior of materials usually ends when the stress increases beyond the so-called yield point (σ_y_) and the material demonstrates plastic behavior, which means it cannot return to its original shape. Figure 1 shows the schematics of the stress–strain curve of a representative material under loading. Increasing the magnitude of applied stress ends in material failure, or fracture, as it is commonly called in the bone research domain. The highest stress applicable on the unit of material before fracture is called ultimate stress (σ_u_). Different mathematical models have been proposed to define the elastic–plastic behavior of bone accurately.

## 4. FE Modeling Principles

**Figure 2:**
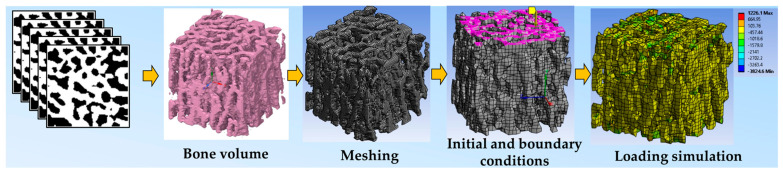
Workflow diagram of micro-CT-based FEA on an exemplary 5 mm^3^ cube of trabecular bone. FEA workflow starts with segmented micro-CT images, followed by meshing, material property, and initial and boundary condition definition (e.g., elements on the top side of the cube, in purple, are selected to apply compressive loading), and concludes with loading simulation and reporting predicted mechanical properties (normal mechanical stress in this example).

Figure 2 shows the workflow diagram of micro-CT-based FEA on an exemplary trabecular bone section. The FEA workflow starts with segmented micro-CT images, followed by meshing, material property, and initial and boundary condition definition, and concludes with loading simulation and reporting predicted mechanical properties. In addition to the selection of the elastic–plastic behavior of the material in FE models, we need to determine several material properties, including the density, E, υ, σ_y_, and σ_u_. This study is focused on µFEA, which basically considers solid elements only on pure bone voxels, assigning the bone marrow and intertrabecular space as background.

The FE method is a numerical simulation involving partial differential equations (PDEs), which should be solved within elements using boundary and initial conditions [14]. After generating the structure from imported 3D images, the first step is meshing, which involves dividing the entire structure into smaller, simpler pieces called elements. These elements can be triangles, squares, tetrahedrons, or other shapes depending on the simulated structure geometry [15]. By discretizing the domain, we essentially convert the continuous problem described by PDEs into a system of algebraic equations. Each element is assigned a set of shape functions that define the variation in physical quantity (i.e., displacement in the current review) within the element. The next step involves transforming the governing PDEs into weak formulations (to avoid solving higher-order derivatives directly) within elements relating the unknown quantities (like displacement at each node) to the known loads and boundary conditions. The element equations from all the individual elements are then assembled into a global system of equations to be solved using the global boundary and initial conditions via various numerical methods like Gaussian elimination and iterative techniques.

## 5. FEA of Animal Models

Table 2 presents a summary of the micro-CT-based FEA of trabecular and cortical bone in animal models, including ex vivo and in vivo studies.

### 5.1. Using FEA to Examine Impacts of Therapies

Several investigations have used micro-CT-based FEA with animal models to evaluate the impact of different treatments on bone formation or loss, as indicated by changes in estimated mechanical properties.

Kim et al. [11] evaluated rat vertebral trabecular bone changes in response to combined dynamic compressive loading stimulation and parathyroid hormone (PTH) therapy (an anabolic treatment to increase bone formation) using micro-CT-based FEA. Their analysis focused on trabecular bone in the proximal, center, and distal regions of vertebral body, by exploring changes in FEA-based mechanical tensors such as principal stress and strain, as well as strain energy density. These regions were defined to localize mechanical responses to the applied loading and treatment conditions. Combined daily mechanical loading and PTH administration significantly increased the bone formation rate compared with the control, as shown by the greater mineral apposition rate and labeled bone surface. Interestingly, positive significant correlations were observed between the bone formation indices and trabecular bone tissue mechanical tensors, likely implying the potential of FEA tensors to predict local bone formation. The effects of various PTH treatments were also examined in other animal model studies. Rhee et al. [14] examined the PTH treatments that resulted in improvements in the microstructural and mechanical properties of bone in vertebral bodies in an osteoporotic rat model after ovariectomy. They showed the feasibility of using micro-CT-based FEA to accurately reflect the differences between the therapeutic efficacy of different treatments in small-animal models.

Liu et al. [31] determined the utility of micro-CT-based FEA in assessing periarticular bone changes in a rabbit model of post-traumatic osteoarthritis treated with risedronate, a drug in the bisphosphonate family. FEA was employed to quantify the mechanical properties and microstructural integrity of femoral condyle bone, revealing significant degradation in untreated animals and the bone-preserving effects of the drug in the treated models. The results underscore FEA’s ability to detect early-stage changes in bone architecture and strength, providing valuable insights into the therapeutic impact of interventions on post-traumatic osteoarthritis progression. Shahnazari et al. [15] investigated the effects of bisphosphonates and their dose on the microstructural and mechanical properties of the vertebral bone in aged osteogenic rat models using micro-CT-based FEA. They revealed that bisphosphonate treatment preserved bone mass and improved vertebral architecture and stiffness, although the higher doses did not significantly improve the localized bone mechanical properties like the elastic modulus and toughness. Similar micro-CT-based FEA has been used recently [17] to investigate the bisphosphonate impact on femoral condyle bone mechanical properties in ovariectomized osteoporotic rat models. Bone stiffness demonstrated a significant increase in addition to standard bone microstructural improvements in treated animals.

In addition to bisphosphonates and PTH, the impact of other osteoporosis treatments on animal models’ bone mechanical properties has also been studied with micro-CT-based FEA. Cabal et al. [29] evaluated the efficacy of odanacatib treatment (cathepsin K inhibitor) in improving bone mineral, microarchitectural, and mechanical properties at the ultra-distal radius of ovariectomized monkeys. High-resolution CT-based FEA was performed longitudinally on the in vivo data. Improvements in cortical thickness with odanacatib were superior to those observed with alendronate (from the bisphosphonate family), resulting in higher estimated bone strength in FEA analyses. These findings support the translational feasibility of using high-resolution CT-based FEA for the in vivo monitoring of bone strength under different treatments. They later validated their results using micro-CT-based FEA and experimental tests on ex vivo data from dissected specimens [24]. Enhanced mechanical properties were reported in the vertebral trabecular bone of monkeys treated with odanacatib. Compressive mechanical tests were performed to measure the apparent elastic modulus of the trabecular cores, while micro-CT-based FEA was used to estimate trabeculae-level elastic modules, which were found to be higher in treated models.

Spatz et al. [28] assessed the effect of unloading and sclerostin antibody (SclAb) treatment on the mechanical properties of metaphyseal bone in mice using micro-CT-based FEA. Midshaft femoral strength, assessed by three-point bending tests, and distal femoral strength, assessed by micro-CT-based FEA simulating compression tests, were significantly higher in the treated group compared to controls. These results suggest that SclAb therapy can mitigate the skeletal deterioration caused by mechanical unloading, demonstrating its potential therapeutic benefit.

**Figure 3:**
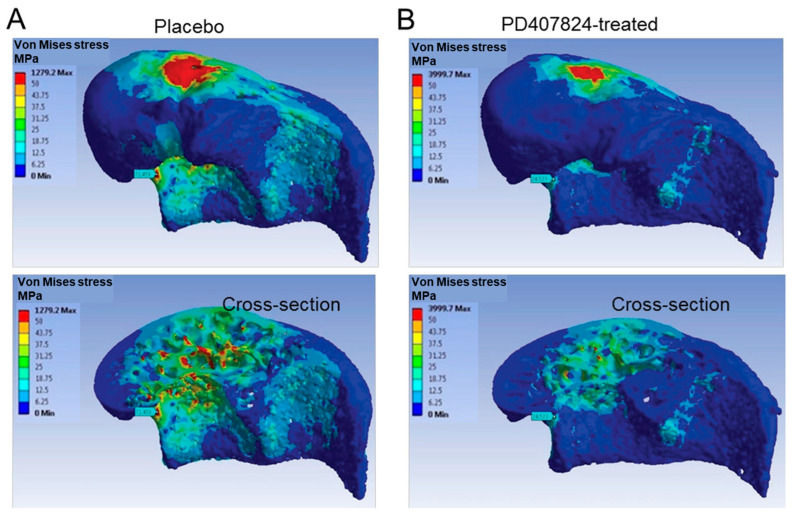
Micro-CT-based von Mises stress distribution color maps in the distal femoral bone of representative mice affected by bone tumor after (**A**) placebo treatment and (**B**) a special treatment (i.e., PD407824). The first row shows a surface stress map, and the second row shows a cross-sectional view of the 3D stress map. The special treatment resulted in more uniform stress distributions, implying its effectiveness. This figure was previously presented by Jiang et al. [23] Reprinting permission was granted based on Creative Commons use guidelines (https://creativecommons.org/licenses/by/4.0/, (accessed on 1 April 2025)). Minor cropping and adjustment has been applied for clarification.

Jiang et al. [23] analyzed a tumor drug’s effect on the mechanical properties of mouse trabecular bone using micro-CT FEA. Their FEA results revealed that treated bones exhibited reduced mechanical stress levels compared to the placebo-treated control, indicating improved resistance to fracture. This study underscores the potential role of micro-CT-based FEA in evaluating the mechanical outcomes of new pharmacological interventions. Figure 3 shows the von Mises stress distribution color maps in the distal femoral bone of representative mice affected by a bone tumor, which was treated with a placebo (Figure 3A) and a proposed new drug (i.e., PD407824, Figure 3B). The stress maps demonstrate a more uniform distribution after the treatment both on the bone surface and a cross-sectional view [23].

The most recent report of micro-CT-based FEA in mice models was investigating the impact of simvastatin, a cholesterol control drug, on vertebral bone mechanical properties in ovariectomized osteoporotic mice models [16]. Vertebral bone stiffness demonstrated significantly higher values, coupled with standard bone microstructural improvements in treated animals.

### 5.2. Using FEA to Examine Impacts of Implants and Surgical Interventions

**Figure 4:**
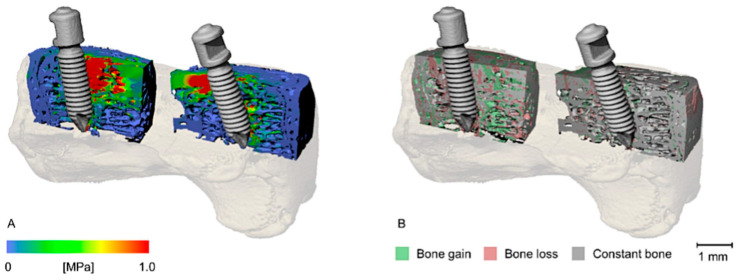
(**A**) Micro-CT-based von Mises stress distribution and (**B**) bone remodeling around the two mini-implants within a rat tail vertebra. Local high stresses were associated with more bone remodeling around the implants. This figure was previously presented by Kerberger et al. [35]. Reprinting permission was granted based on Creative Commons use guidelines (https://creativecommons.org/licenses/by/4.0/, (accessed on 1 April 2025)).

A limited number of studies have used FEA to investigate the impacts of implants and surgical interventions on bone mechanical performance. Jaecques et al. [34] employed micro-CT-based FEA to predict stress and strain states in guinea pigs’ tibial bone after placing percutaneous implants. The study utilized a computer-aided developed model of the implant, which was created independently from the in vivo animal images and based on the correct implant dimensions (derived from a separate µCT scan of the implant). This study demonstrated the feasibility of using micro-CT-based FEA to predict peri-implant bone adaptation in order to optimize porous bone scaffold designs. Kerberger et al. [35] used micro-CT-based FEA to study implant migration and associated bone remodeling in rat tail vertebrae subjected to constant forces, mimicking orthodontic mini-implants connected with contraction springs. FEA-based mechanical stress values tended to increase for two weeks post-implantation and decrease thereafter. As shown in Figure 4, bone regions between the implants subjected to loading experienced higher von Mises stress values, which resulted in bone remodeling involving initial bone loss within the first two weeks and a subsequent bone gain up to week eight [35].

### 5.3. Using FEA to Investigate Mechanical Loading Effects and Bone Fracture Mechanisms

A few studies have used micro-CT-based FEA as a tool to better understand the bone fracture mechanism and the mechanical loading impact on the bone remodeling. Nagaraja et al. [33] utilized micro-CT-based FEA to analyze the deformation and damage mechanisms in bovine trabecular bone under uniaxial compression. By combining sequential fluorescent staining with micro-CT-based FEA, they studied the relationship between the mechanical stresses and the regions of trabecular bone microdamage. Sequential fluorescent staining enabled them to track the progression of deformation in bone elements. The analysis revealed that microdamage accumulation began at regions experiencing stresses and strains lower than previously reported for the mechanical yielding of trabecular bone. This finding underscores the role of cumulative microdamage in skeletal fragility and highlights the relevance of FEA in predicting bone fracture. Furthermore, the results align with prior research [28], which demonstrated significant correlations between regions stimulated by higher mechanical stresses or strains and increased bone remodeling.

Bone remodeling is believed to be orchestrated with internal and external loading, potentially after bone microdamage. Ju et al. [21] evaluated the effects of jumping and running exercises on rat trabecular bone architecture and strength using micro-CT-based FEA. They revealed that both running and jumping exercises significantly increased bone strength compared to controls, as estimated by FEA fracture loads. However, no significant difference was observed between the two groups. Running exercise predominantly enhanced the trabecular number, while jumping exercise primarily increased trabecular thickness, suggesting distinct adaptive mechanisms underlying these exercise modalities.

Fan et al. [25] developed a multiscale and biphasic FEA framework to predict the local mechanical responses of mouse bone to loading, such as strain distribution, interstitial fluid pressure, and shear forces within the bone tissue. The whole tibial bone, including the cortical and trabecular bone, was modeled using linear elastic FEA, and the matrix deformations at various locations were calculated under axial loading. A segment of the whole-bone model was then imported to the biphasic poroelasticity analysis platform to predict load-induced fluid pressure fields and interstitial solute flows. Notably, the segment used in the biphasic analysis was imaged using a confocal microscope with a ~0.2-micrometer voxel size. Such a multiscale and biphasic FEA framework can help in understanding potential cellular mechanisms underlying the anabolic power of exercises and physical activities in bone recovery.

Modeling bone structures within their anatomical context is critical to accurately capture realistic mechanical behaviors. Harrison and McHugh [32] investigated the mechanical behavior of ovine trabecular bone in isolated core samples compared with whole vertebrae bodies, and observed significant variation in apparent core modulus values and strain distributions.

### 5.4. Miscellaneous Bone FEA Studies

Employing micro-CT-based FEA has not been limited to the abovementioned studies. For example, Liu et al. [30] investigated site-specific changes in bone quantity and quality during and after lactation using a combination of micro-CT-based microstructural assessment and micro-FEA. This approach provided a noninvasive estimation of bone stiffness, which closely correlates with bone strength (positive relationship) and fracture risk (negative relationship). Their analysis revealed that lactation leads to significant bone loss and reduced bone mechanical stiffness, with partial recovery post-weaning. Whole-bone stiffness in the lumbar spine was reduced by 71% in lactating mice compared to nulliparous controls, but this difference diminished in recovered mice. In contrast, the stiffness at the proximal tibia and distal femur remained significantly lower in recovered mice compared to controls. These findings highlighted significant differences between lactating and nulliparous mice in trabecular microarchitecture and tissue mineralization being more resilient in some regions than others.

In another miscellaneous study, Wu et al. [18] utilized micro-CT-based FEA to evaluate the effects of ionizing radiation on murine lumbar vertebrae. The study revealed that ionizing radiation negatively affected trabecular microarchitecture and bone volume, increasing reliance on the vertebral cortex for axial load resistance. Despite these structural changes, FEA results indicated that the vertebral cortex’s load-bearing capacity remained stable during the short-term follow-up period.

Micro-CT-based FEA was also used to evaluate the mechanical properties of bovine femoral trabecular bone along different anatomical orientations by Lin et al. [27]. Stiffness values from FEA and ex vivo mechanical testing showed no significant differences between orientations. It should be noted that other anatomical sites or animal models may not be comparable to this study. When pooling stiffness data across orientations, a strong correlation was observed between FEA prediction and experimental results, further supporting the validity of the method for biomechanical analyses. In a different study, Huang et al. [19] used micro-CT-based FEA and experimental tests on 3D-printed specimens of rat bone with similar structures but differing BV/TV to confirm the long-known power law relationship between mechanical properties and BV/TV.

## 6. Continuous Improvements in FEA

Several investigators have been proposing novel approaches to improve the accuracy of FE models while reducing processing time and computational resource requirements. Wu et al. [26] suggested the selection of the volume of interest for FEA to be focused only on the regions experiencing more geometrical differences in rat bone based on the micro-CT images. This approach effectively utilizes computational resources and shortens the data processing time. Santaella and Tseng [22] demonstrated that an element reduction approach constitutes a valid protocol to simulate trabecular bone porosity in carnivorans. This alternative approach for incorporating porosity in FE models offers a fast and effective way to approximate trabecular geometry without relying on high-resolution scans. They conclude that maintaining or mimicking the internal porosity of a trabecular structure is a more effective method of approximating trabecular bone behavior in FE models than modifying material properties.

Du et al. [20] evaluated the effects of inhomogeneous material property distribution in simulating trabecular bone remodeling in bovine femurs. Their two-phase FE model demonstrated that initial material distributions, whether multi- or single material, resulted in similar inhomogeneous density distributions, though subtle morphological differences were noted. Bone region inhomogeneous material properties such as Young’s modulus and BMD were calculated according to the gray-scale value distribution in images. The non-bone region was assigned a very low module of elasticity—3 MPa—with a Poisson’s ratio of 0.17, which had a negligible effect on the overall structural integrity of the trabecular structure. These findings suggest that simplified models may still yield realistic predictions, but with limitations in capturing finer structural details and local mechanical properties.

Gerasimov et al. [36] applied micro-CT-based FEA to simulate three-point bending in porous pig bone structures. A three-dimensional iso-parametric finite element of a continuous medium was developed by the authors with a linear approximation, based on weighted integration of the local stiffness matrix. They described a general algorithm for constructing a numerical model that allows the static calculation of objects with a porous structure according to its micro-CT data. The method effectively captured stress–strain distributions and aligned closely with experimental results. This validation highlights the efficacy of FEA for analyzing complex porous structures in animal models.

## 7. Discussion

In this study, we reviewed the methods and results of micro-CT-based FEA studies performed on animal models. Micro-CT-based FEA evaluates the impact of different therapeutics on bone mechanical competence, which is not only determined by the standard bone microstructural parameters, such as BV/TV or trabecular thickness, but also by the 3D connectivity of the bone structure and its material distribution, as well as the loading magnitude, direction, and scenarios.

Similar to standard microarchitectural parameters, FEA results rely on high-quality trabecular bone segmentation, which in turn can be affected by original image quality, primarily spatial resolution, the contrast-to-noise ratio, and often-seen artifacts described in previous studies [37]. Comparing the available micro-CT voxel size and the typical trabecular bone thickness in target animals is critical to decide on the accuracy of µFEA, such that a minimum of 10 µm voxel size is required to run an accurate µFEA in rodents. However, for low-resolution images, macro-scale or continuum-space FEA analysis can be recommended.

The use of micro-CT-based FEA in the literature has been mainly focused on evaluating the changes in the mechanical properties of animal model bones as a result of different treatments, such as bisphosphonates [31], PTH [11], and SclAb [28]. Micro-CT-based FEA has also been used to study the bone fracture mechanism and the impact of mechanical forces on bone remodeling [21,25,33]. These studies indicate that micro-CT-based FEA not only enhances our understanding of fracture mechanisms but also holds potential for individualized fracture risk assessment and the development of novel therapeutic strategies.

One of the main potential applications of micro-CT-based FEA is investigating the impacts of implants and surgical interventions on bone mechanical performance; however, only a few studies relating to this have been reported in the literature to the authors’ knowledge [34,35]. For example, micro-CT-based FEA has been used to analyze stress distribution in guinea pig tibiae following percutaneous implant placement and demonstrated that implant shape and positioning are key determinants of bone remodeling patterns [34]. Studies of FEA-based stress distribution around continuously loaded implants have shown that FEA can predict regions undergoing larger remodeling [35]. This application will be invaluable in human studies, where experimenting with new implants is restricted.

Novel approaches to improve the accuracy of micro-CT-based FEA have been sought by several research groups in order to reduce the time and computational resource requirements. Recent studies have introduced region-specific modeling [26], focusing the model on regions with distinct microstructural differences, and used multi-scale simulations [25] as innovative strategies to enhance computational efficiency while maintaining predictive accuracy. In parallel, machine learning (ML)-assisted image reconstruction and segmentation [38,39,40] methods have been developed that can improve the segmented bone volume imported into FE models. Improving and accelerating micro-CT-based FE models with ML methods, such as applying ML-driven meshing algorithms and material property estimation, may be a practical future direction that has not been explored or reported in the literature, to the authors’ knowledge.

Notably, validation is necessary for any developed FE model, regardless of the utilized imaging modality or data processing steps. The reliability of FEA strongly depends on the accuracy of the material properties assigned to the elements of the model, as well as the boundary and loading conditions applied. FE model validation is often performed via comparisons between the simulated results and an equivalent mechanical experiment or previously validated models. For example, Cabal et al. demonstrated strong correlations between FEA-estimated mechanical properties and ex vivo biomechanical compression tests in primates, supporting the validity of micro-CT-based FEA in predicting bone strength [24,29,36]. Standardized validation protocols should be established to ensure reproducibility across different studies. Simulating standard compression and performing tensile tests on plugs of bone, following the American Society for Testing and Materials (ASTM) recommendations [41], are likely practical validation methods.

In addition to these advancements, micro-CT-based FEA holds significant promise for clinical translation. The development of patient-specific FEA models, based on in vivo imaging and ML-assisted simulations, could bridge the gap between preclinical animal studies and human applications. Such models could aid in personalized treatment planning for osteoporosis, bone fractures, and implant design, ultimately enhancing clinical decision-making. Despite the advantages of micro-CT-based FEA, several challenges remain, including high computational costs, model complexity, and the need for standardized validation protocols. Future studies should focus on refining computational models, validating their predictive accuracy through experimental testing, and developing patient-specific FEA approaches based on in vivo imaging data. By addressing these challenges, micro-CT-based FEA will continue to advance as a crucial technique for understanding bone biomechanics, ultimately bridging the gap between animal research and clinical applications in osteoporosis treatment, implant design, and fracture prevention.

## 8. Conclusions

Micro-CT-based FEA has emerged as a powerful tool for assessing bone mechanical properties and evaluating the effects of pharmacological treatments, implants, and surgical interventions in animal models. This review summarizes the results of reported micro-CT-based FEA studies in the literature. Moreover, the steps of FE development, basic concepts of mechanical properties required for understanding FEA results, limitations, and future potential of micro-CT-based FEA have been discussed. Accurate micro-scale FEA can be performed when imaging voxel size is fine enough to resolve the typical trabecular bone thickness in target animal models. The future integration of ML is recommended to make FEA faster and more accurate.

## Figures and Tables

**Table 2:** Summarized micro-CT-based FEA of trabecular and cortical bone in animal models (ex vivo and in vivo). For all reviewed studies, the element material was defined as homogeneous, linear, and isotropic. Poisson’s ratio was equal to 0.3 in all summarized studies.

First Author, Year	Micro-CT Voxel (Element Size)	Element Properties	Finite Element Software	Ex/In Vivo, Bone Site
Liang et al., 2025 [16]	15 µm^3^	Hexahedron element Elastic modulus, E = 15 GPa	ANSYS, Ver 17	- ex vivo - mice vertebra - trabecular
Wang et al., 2024 [17]	10 µm^3^	Quadratic tetrahedron element E = 13 GPa	ABAQUS Ver 2020	- ex vivo - rat femoral condyle - trabecular
Wu et al., 2023 [18]	10 µm^3^	Eight-noded brick element E = 10 GPa	custom-written code	- ex vivo - mice vertebrae - trabecular
Huang et al., 2023 [19]	15 µm^3^	Tetrahedral element E = 3 GPa	custom-written code	- ex vivo - rat femur - trabecular
Du et al., 2020 [20]	60 µm^3^	Tetrahedral element E = 12 GPa	ABAQUS Ver 6.4	- ex vivo - bovine femoral head - trabecular
Ju et al., 2020 [21]	18 µm^3^	Not mentioned	TRI/3D-FEM64 (Version not reported)	- ex vivo - rat femur - trabecular
Santaella et al., 2019 [22]		Tetrahedral element E = 20 GPa	Strand 7 Ver 2.4.6	- ex vivo - carnivoran - trabecular
Jiang et al., 2018 [23]	9 µm^3^	Tetrahedral element E = 8.9 GPa	ANSYS Ver 14.5	- ex vivo - mouse distal femur - trabecular
Cabal et al., 2017 [24]	20 µm^3^	Tetrahedral element E = 12 GPa	ANSYS (Version not reported)	- ex vivo - monkey vertebra - trabecular
Fan et al., 2016 [25]	20 µm^3^	Tetrahedral element E = 20 GPa	Altair HyperWorks (Version not reported)	- in vivo - mouse tibia - cortical and trabecular
Wu et al., 2015 [26]	18 µm^3^	Hexahedral element E = 24.5 GPa	ABAQUS Ver 6.10	- ex vivo - rat lumbar vertebra - trabecular
Lin et al., 2014 [27]	18 µm^3^	Tetrahedral element E = 15.9 GPa	ABAQUS Ver 6.10	- ex vivo - bovine femur - trabecular
Spatz et al., 2013 [28]	12 µm^3^	E = 10 GPa	Scanco Medical AG (Version not reported)	- ex vivo - mice tibia - trabecular and cortical
Cabal et al., 2013 [29]	41 μm^3^	E = 18 GPa	ANSYS (Version not reported)	- ex vivo - monkey - trabecular and cortical
Liu et al., 2012 [30]	10.5 µm^3^	Eight-noded brick element E =15 GPa	custom-written code	- ex vivo - mice tibia, spine, femur - trabecular
Liu et al., 2012 [31]	38 µm^3^	Hexahedron element E = 15 GPa	custom-written code	- ex vivo - rabbit femur - trabecular
Harrison et al., 2010 [32]	36 µm^3^	Tetrahedral element E = 8.5 GPa	custom-written code	- ex vivo - ovine vertebra - trabecular
Shahnazari et al., 2010 [15]	10.5 µm^3^	Eight-noded prismatic E = 18 GPa	custom-written code	- ex vivo - rat lumbar vertebra - trabecular and cortical
Rhee et al., 2009 [14]	21.3 µm^3^	Hexahedron element E = 1 GPa	ANSYS Ver 09	- ex vivo - rat vertebra - trabecular
Nagaraja et al., 2005 [33]	20 µm^3^	Hexahedral element E = 18.4 GPa	Scanco Medical AG (Version not reported)	- ex vivo - bovine proximal tibia - trabecular
Jaecques et al., 2004 [34]	15.9 µm^3^	Tetrahedral element E = 11 GP	MSC Nastran/Pattern (Version not reported)	- in vivo - guinea pig tibia - trabecular
Kim et al., 2003 [11]	34 µm^3^	Eight-noded brick element E = 18 GPa	custom-written code	- ex vivo - rat tail vertebra - trabecular
